# Reduction of voltage gated sodium channel protein in DRG by vector mediated miRNA reduces pain in rats with painful diabetic neuropathy

**DOI:** 10.1186/1744-8069-8-17

**Published:** 2012-03-22

**Authors:** Munmun Chattopadhyay, Zhigang Zhou, Shuanglin Hao, Marina Mata, David J Fink

**Affiliations:** 1 Department of Neurology, University of Michigan and VA Ann Arbor Healthcare System, Ann Arbor, MI, USA; 2 1500 E Medical Center Drive, Ann Arbor, MI 48109, USA; 3 Department of Pathology, University of North Carolina, Chapel Hill, NC, USA; 4 Department of Anesthesiology, University of Miami, Miami, FL, USA

**Keywords:** Diabetes, Pain, Neuropathy, Gene therapy, Sodium channel

## Abstract

**Background:**

Painful neuropathy is a common complication of diabetes. Previous studies have identified significant increases in the amount of voltage gated sodium channel isoforms Na_V_1.7 and Na_V_1.3 protein in the dorsal root ganglia (DRG) of rats with streptozotocin (STZ)-induced diabetes. We found that gene transfer-mediated release of the inhibitory neurotransmitters enkephalin or gamma amino butyric acid (GABA) from DRG neurons in diabetic animals reduced pain-related behaviors coincident with a reduction in Na_V_1.7 protein levels in DRG *in vivo*. To further evaluate the role of Na_V_α subunit levels in DRG in the pathogenesis of pain in diabetic neuropathy, we constructed a non-replicating herpes simplex virus (HSV)-based vector expressing a microRNA (miRNA) against Na_V_α subunits.

**Results:**

Subcutaneous inoculation of the miRNA-expressing HSV vector into the feet of diabetic rats to transduce DRG resulted in a reduction in Na_V_α subunit levels in DRG neurons, coincident with a reduction in cold allodynia, thermal hyperalgesia and mechanical hyperalgesia.

**Conclusions:**

These data support the role of increased Na_V_α protein in DRG in the pathogenesis of pain in diabetic neuropathy, and provide a proof-of-principle demonstration for the development of a novel therapy that could be used to treat intractable pain in patients with diabetic neuropathy.

## Background

Pain is a common complication of diabetic neuropathy that, despite substantial advances in understanding of pathophysiology, remains relatively refractory to treatment with available agents [1]. In rats with streptozotocin (STZ) induced diabetes and painful neuropathy, an increase in the alpha (pore-forming) subunit of voltage gated sodium channel isoform 1.7 (Na_V_1.7) in primary sensory afferent neurons of the dorsal root ganglia (DRG) has been reported [2], a change that correlates with increased amplitude and negative shift of the activation of tetrodotoxin (TTX)-sensitive current in those neurons. A potential pathogenic role for Na_V_1.7 in the development of pain in this syndrome is supported by the observation that gain of function mutations in Na_V_1.7 cause inherited spontaneous neuropathic pain syndromes primary erythermalgia [3,4] and paroxysmal extreme pain disorder [5].

In previous studies we have constructed a series of herpes simplex virus (HSV)-based gene transfer vectors that effectively transduce DRG *in vivo *from skin inoculation, and have used these vectors to express inhibitory neurotransmitters [6-8] or neurotrophic factors [9-11]. In order to explicitly test the role of increased levels of Na_V _in DRG in the pathogenesis of pain in PDN, we constructed a non-replicating herpes simplex virus (HSV)-based vector to reduce Na_V_α protein in DRG, and compared the effect of Na_V_α subunit knockdown on pain-related behaviors in PDN with the effect in a standard model of inflammatory pain.

## Results and discussion

The data reported here demonstrate that, 1) an HSV vector expressing an miRNA against voltage gated Na_V _alpha subunits reduces expression of Na_V_s in DRG *in vivo*; 2) normalization of Na_V_1.7 levels in STZ-diabetic rats achieved by the miNa_V_-expressing vector substantially reduced pain related behaviors in the STZ rat model of painful diabetic neuropathy; but in comparison, 3) the reduction in expression achieved by the miNa_V_-expressing vector produced only a modest reduction in inflammatory pain (flinching) in the acute and delayed phases of the formalin test.

### Knockdown of Na_V_α channels

**Figure 1:**
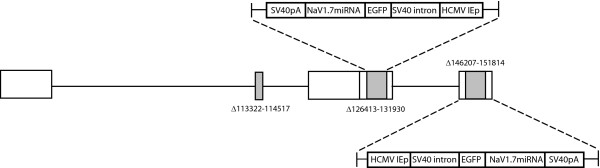
**Vector schematic of QHmiNa_V_**. QHmiSc is identical except that the specific Na_V_1.7 miRNA sequence is replaced by a scrambled sequence.

A series of miRNA sequences targeting common rat Na_V _α subunits were constructed and inserted into the nonreplicating HSV recombinant UL41E1G6-M [12]. The resulting series of vectors were used to transfect primary DRG neurons in culture at a multiplicity of infection (MOI) of 1 for 2 hours, and 48 hours later the amount of Na_V_1.7 mRNA determined by RT-PCR. The most effective vector construct, designated QHmiNa_V _(Figure 1) was used in the experiments reported. Control vector QHmiSc was identical to QHmiNa_V _but contained a scrambled sequence in place of Na_V _miRNA sequence.

**Figure 2:**
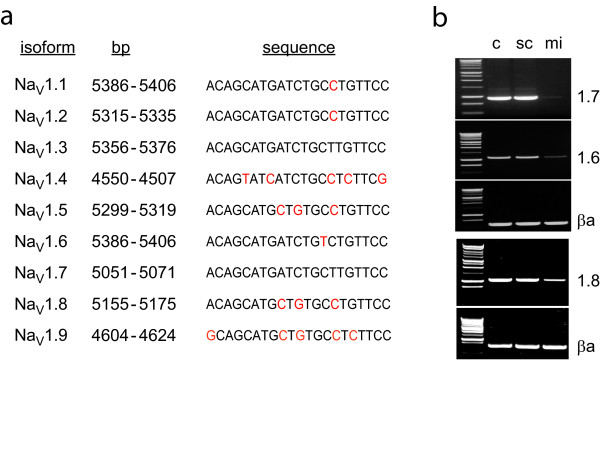
**a. Comparison of the sequence used for the miRNA construct (Na_V_1.7) with other Na_V _alpha isoforms**. Mismatched base pairs are indicated in red. b. RT-PCR of Na_V _alpha subunit isoforms from primary DRG neurons in vitro infected with QHmiSc (sc) or QHmiNa_V _(mi). c = uninfected cells; βa = β-actin.

The sequence inserted into QHmiNa_V _was a perfect match for Na_V_1.7 and for Na_V_1.3, but an imperfect match for other Na_V_s (Figure 2a). We examined the effect of QHmiNa_V _infection on expression of DRG Na_V _isoforms Na_V _1.3, 1.6, 1.7 and 1.8 by examining isoform- specific mRNA levels 3 days after infection of primary DRG neurons in culture at an MOI of 1. Infection with QHmiNa_V _produced a substantial reduction in the levels of Na_V_1.6 and 1.7 mRNAs, and about a 50% reduction in the amount of Na_V_1.8 mRNA in infected DRG neurons (Figure 2b). Infection with QHmiSc at an MOI of 1 resulted in no change in Na_V _α subunit mRNA levels. The change in Na_V _protein levels assessed using a well characterized anti-Na_V_1.7 antibody, lagged several days behind the reduction in RNA reaching 80% of basal levels at 10 days after infection reflecting the half-life of already synthesized protein (data not shown).

Na_V_s are crucial determinants of neuronal excitability [13,14], and the Na_V _isoforms Na_V_1.3, Na_V_1.6, Na_V_1.7, Na_V_1.8, and Na_V_1.9 are all expressed in DRG neurons. Transgenic mouse and knockdown studies have principally implicated the isoforms Na_V_1.3, Na_V_1.8 and Na_V_1.9 in inflammatory and nerve injury related pain [15-19]. Na_V_1.8-null mutant mice lack slowly inactivating sodium currents and unable to sense cold pain or mechanical pressure [20]. These animals also exhibit deficits in inflammatory pain behavior, yet they respond normally to heat. In carrageenan and complete Freund's adjuvant-induced hyperalgesia there is an increase in Na_V_1.8 and Na_V_1.9 currents [21,22] and an increase in the expression of Na_V_1.8 in DRG has been reported [23]. Knockdown of Na_V_1.8 by intrathecal delivery of antisense oligonucleotide against Na_V_1.8 reduces CFA-induced hyperalgesia [24,25].

### QHmiNa_V _reverses the increase in Na_V_1.7 caused by diabetes

**Figure 3:**
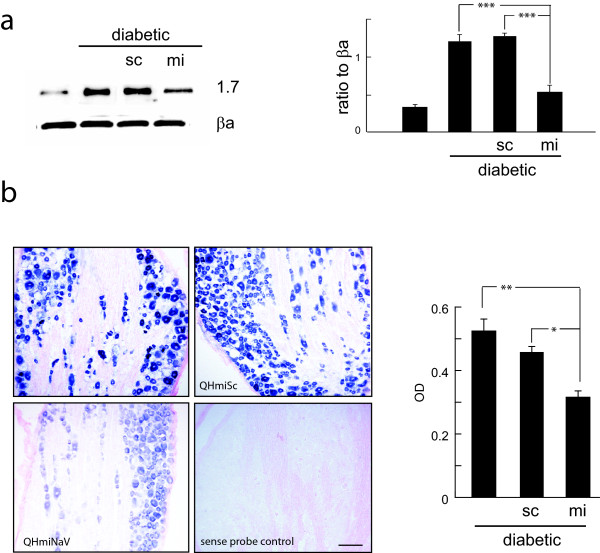
a. Na_V_1.7 levels in DRG of diabetic animals inoculated with QHmiNa_V _demonstrates reduction in protein compared to QHmiSc 4 weeks after inoculation (*** *p *< 0.005). Data presented as ratio to β-actin (βa). b. In situ hybridization using a probe specific for Na_V_1.7 in DRG from diabetic animals, without treatment or inoculated with QHmiSc or QHmiNa_V _as indicated. A sense probe showed no staining. The average optical density of DRG neurons in each condition was determined using a PC based image analysis program (MCID). * *p *< 0.05; ** *p *< 0.01.

There is a significant increase in Na_V_1.7 protein in DRG of rats rendered diabetic by injection of streptozotocin (STZ) [2,6] that correlates with thermal hyperalgesia, mechanical hyperalgesia and cold allodynia, characteristic of painful diabetic neuropathy in these animals. Subcutaneous inoculation of HSV vectors results in transduction of ipsilateral DRG [26]. Two weeks after the onset of diabetes 30 μl containing either 3 × 10^9 ^pfu of QHmiNa_V _or QHmiSc was injected into the plantar surface of both hind feet. Two weeks after the vector inoculation (4 weeks after onset of diabetes), the amount of Na_V_1.7 protein was significantly increased in the diabetic animals (Figure 3a) and in diabetic animals inoculated with the QHmiSc, but there was a substantial reduction in the amount of Na_V_1.7 in diabetic animals inoculated with QHmiNa_V _(Figure 3a). QHmiNa_V _brought the amount of Na_V_1.7 protein in diabetic animals back to near normal but not completely normal levels. By *in situ *hybridization the reduction in Na_V_1.7 RNA expression was found to be widely distributed in neurons in the DRG, and the in situ study suggested a reduction of about 50% in the amount of Na_V_1.7 mRNA in the DRG compared to diabetic animals (Figure 3b). To separately estimate the number of DRG neurons transfected after footpad inoculation of the vector, we performed *in situ hybridization *for the reporter gene GFP which revealed 40-50% of the neurons in individual sections with GFP reporter RNA (Additional File 1).

### HSV-mediated knockdown of Na_V _ameliorates pain-related behaviors in diabetic animals

**Figure 4:**
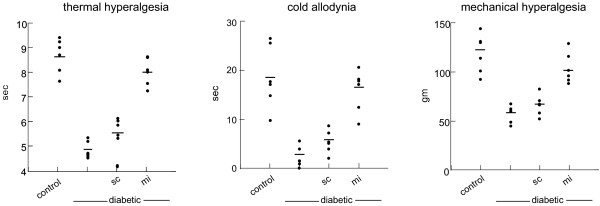
**Pain-related behaviors in diabetic animals inoculated with QHmiNa_V _were marked improved compared to animals inoculated with QHmiSc: thermal latency (left panel); cold latency (middle panel); mechanical threshold (right panel)**. Individual data points are presented; horizontal line indicates mean value. Statistical significance is reported in the results.

Diabetic rats demonstrate thermal hyperalgesia, cold allodynia and mechanical hyperalgesia [6,8]; all of these signs of neuropathic pain were improved by inoculation with QHmiNa_V_. Thermal hyperalgesia was manifested by a decrease in withdrawal latency in response to noxious thermal stimuli (control 8.62 ± 1.3 sec; diabetic 4.89 ± 0.5 sec; *p *< 0.005). Two weeks after inoculation (4 weeks after diabetes) animals inoculated with QHmiNa_V _showed a statistically significant increase in thermal latency (QHmiNa_V _7.92 ± 0.7 sec compared to diabetic; *p *< 0.005) and control vector QHmiSc inoculated diabetic animals (QHmiSc 5.5 ± 0.8 sec; *p *< 0.01 compared to QHmiNa_V_) (Figure 4). Cold allodynia was manifested by a decreased latency to withdraw from a cold acetone spray in diabetic compared to control animals (diabetic 3.1 ± 0.6 sec; control 18.1 ± 3.9 sec *p *< 0.001). Diabetic animals inoculated with QHmiNa_V _showed an increased latency to withdraw from this stimulus compared to diabetic control vector inoculated animals (QHmiNa_V _16.9 ± 2.1 sec; QHmiSc 6.2 ± 2.1 sec; *p *< 0.001). Mechanical hyperalgesia was tested using the method described by Randall and Sellito. Inoculation of QHmiNa_V _significantly increased the pressure threshold (QHmiNa_V _105.9 ± 4.9 gm) compared to diabetic animals (55.5 ± 3.4 gm; *p *< 0.001) and QHmiSc-inoculated animals (62.7 ± 2.9 gm; *p *< 0.001) measured 4 week after inoculation (Figure 4).

In STZ-induced PDN there is a marked increase in Na_V _1.7 protein in DRG [2,27,28], an increase that is mediated by phosphorylation of PKC [6,8]. While the pathogenesis of pain in PDN is complex and many molecular entities may be involved [29], in studies using HSV-based vectors to transfer genes to the DRG in vivo, we observed that vector-mediated release of gamma aminobutyric acid (GABA) from the DRG of diabetic animals results in a reduction in pain-related behaviors, coincident with a reduction in Na_V_1.7 protein in the DRG achieved through activation of presynaptic GABA_B _receptors [8]. We also found that gene transfer-mediated release of the inhibitory neurotransmitter enkephalin resulted in a greater reduction of neuropathic pain-related behaviors in animals with neuropathic pain resulting from diabetic neuropathy [6] compared to animals with neuropathic pain resulting from spinal nerve ligation [7], and that continuous activation of presynaptic delta opioid receptors by vector-produced enkephalin in the diabetic animals also resulted in a reduction in the amount of Na_V_1.7 protein in diabetic DRG *in vivo*.

These results support the interpretation that increased Na_V_1.7 protein in DRG plays in the pathogenesis of pain in this model of PDN. The potential role of Na_V_1.7 in the pathogenesis of pain in PDN is also supported by the observation that gain-of-function mutations in SCN9A, the gene encoding Na_V_1.7 result in the spontaneous pain syndromes primary erythermalgia and paroxysmal extreme pain disorder [30,31], and that loss of function mutations in Na_V_1.7 result in an inherited channelopathy characterized by total insensitivity to pain [32].

Na_V_1.7 is the principal TTX-sensitive channel in small DRG neurons, and is responsible for almost half of the current in those neurons, and the increase in Na_V_1.7 protein is accompanied by an increase in TTX-sensitive current in those cells [2]. There is also an increase in TTX-resistant currents in diabetic rats [27,33] that is likely related to modulation of individual channel properties related to phosphorylation [2]. Waxman and colleagues have suggested that Na_V_1.7, which opens in response to slow ramp depolarization may serve to "set the gain" for repetitive firing [14]. If that is the case, an increase in the number of Na_V_1.7 channels can serve to lower the gain allowing for altered thresholds and spontaneous pain. There is one early published report in which an increase in Na_V_1.7 in the DRG of STZ-diabetic rats was not observed using immunocytochemistry [27], but several subsequent studies using Western blot found an increase in Na_V_1.7 [2,6,8]. The results of the current study provide support for the interpretation that an increase in the amount of Na_V_1.7 plays a role in the pathogenesis of pain in this model of PDN.

### QHmiNa_V _reduces Na_V_1.7 and 1.8 in normal animals, but produces only a modest reduction in formalin-induced flinching

**Figure 5:**
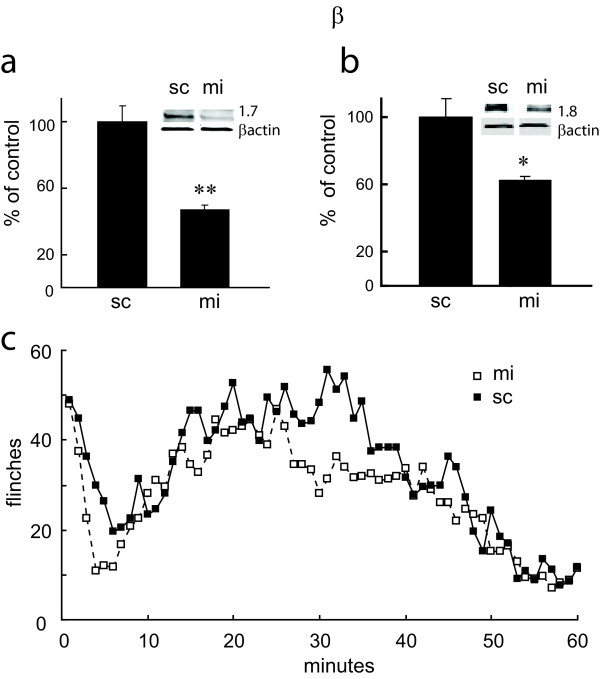
**a. Na_V_1.7 (a) and Na_V_1.8 (b) protein in DRG of animals inoculated with QHmiNa_V _or QHmiSc (sc) 21 days earlier**. Data quantitated as ratio to β-actin and presented as % of control. c. Number of flinches over time after subcutaneous injection of formalin 21 days after inoculation with QHmiNa_V _or QHmiSc.

Twenty one days after subcutaneous inoculation of QHmiNa_V _into the hind paw of normal rats, the amount of Na_V_1.7 was reduced by 50% and the amount of Na_V_1.8 by 40% compared to QHmiSc-inoculated animals (Figure 5a and 5b). Animals inoculated with QHmiNa_V _showed a small but statistically significant reduction in formalin - induced flinching behavior in the delayed phase of the formalin test (Figure 5c).

There is always a possibility in experiments of this type that the directed siRNA may knock down the expression of proteins unrelated to the target gene. While this is an important consideration, the prior evidence indicating a role for Na_V _in the pathogenesis of pain, coupled with the reduction in pain when Na_V _expression is reduced to just that we are looking at on-target effects of Na_V _knockdown. We do not interpret our observation that vector-mediated knock down of Na_V _resulted in only a modest reduction in spontaneous pain in the formalin test to indicate that Na_V_1.7 may not important in inflammatory pain. Yeomans and coworkers reported previously that a related HSV vector engineered to express an Na_V_1.7 antisense construct reduced inflammatory hyperalgesia resulting from injection of complete Freund's adjuvant [34], and Woods and colleagues reported that mice with double knockouts for both Na_V_1.7 and Na_V_1.8 show a substantial reduction in pain related behavior in the delayed phase of the formalin test, in contrast to Na_V_1.8 knockouts that demonstrate a normal behavior in the delayed phase [35]. The results of the current study however do suggest that a 40 to 50% reduction from normal amounts in the amount of Na_V_1.7 and Na_V_1.8 in the DRG does not impair the ability of nociceptors to respond in a functionally meaningful manner to the acute inflammatory stimulus.

Woods and colleagues also reported that the Na_V_1.7 and Na_V_1.8 double knockouts demonstrate no impairment in the development of pain in response to nerve injury (L5 spinal nerve ligation) [35]. Those results, as well as other experiments that demonstrate different changes in Na_V _isoform levels in different models of neuropathic pain [36] are not incompatible with the current study. Rather, they point to what may be important differences in the pathogenesis of different forms of neuropathic pain, in particular the role played by the substantial increase in Na_V_1.7 protein in PDN.

## Conclusions

HSV is a vector that is uniquely suited to deliver genes into DRG neurons in vivo. While much of the previously published work using HSV based vectors has characterized the delivery, expression and biological properties of peptides or proteins expressed from these vectors that are released from transduced neurons [37-39], the results of the current study extend the utility of these vectors to the production of interfering RNAs acting intracellularly. A nonreplicating HSV vector expressing preproenkephalin has been brought to human trial [40]. It is possible that a miRNA-expressing HSV vector could be used in a similar fashion to treat patients with intractable persistent pain from diabetic neuropathy.

## Methods

### Vectors

To construct miRNAs targeting rat Na_V _α subunits present in DRG, we constructed a vector to express a miRNA targeted to a common sequence of Na_V_1 α subunits. A3 top strand oligo 5'-TGC TGG GAA CAA GCA GAT CAT GCT GTG TTT TGG CCA CTG ACA CAG CAT GCT GCT TGT TCC-3' A3 bottom strand oligo 5'-CCT GGG AAC AAG CAG CAT GCT GTG TCA GTC AGT GGC CAA AAC ACA GCA TGA TCT GCT TGT TCC C -3'. Equal amounts of the single-stranded oligos were annealed to generate a double-stranded oligo that ligated with the linearized plasmid pcDNATM6.2-GW/EmGFP-miRNA (Invitrogen), amplified using the following PCR primers with mulI site at 5' end an ECORI site at 3' end, forward primer: 5'-ACG CGT GCT AGT TAA GCT ATC AAC AAG-3' and reverse primer: 5'-GAA TTC GTA CAA GAA AGC TGG GTC TAG-3'. The PCR fragment was extracted by Qia-quick Gel Extraction Kit (Qiagen, Valencia, CA), and 3 ul of the gel-extracted PCR fragment ligated into PGEM-T vector system I kit (Promega; Madison, WI) for expansion. The resulting PCR fragment was cut out from PGEM-T plasmid by MluI and EcoRI, purified by gel electrophoresis and cloned into MluI and EcoRI -cut shuttle plasmid SASB3-M4. The shuttle plasmid containing the insert was then cotransfected with the non-replicating HSV vector UL41E1G6-M in complementing 7B cells, and and a single green virus isolated through serial dilution. Every round of purification was confirmed by sequencing, and the construct designated QHmiNa_V_. A control vector identical to QHmiNa_V_, but containing a scrambled-miRNA sequence in place of Na_V _α subunit miRNA was constructed and designated QHmiSc.

### Diabetic animal model

Following an overnight fast, male Sprague Dawley rats weighing 225-250 gms were injected with streptozotocin (STZ, Sigma, USA) 50 mg/kg, i.p. in citrate buffer (pH 5.5). The development of diabetes was confirmed by measuring blood glucose and animals with blood glucose level ≥ 300 mg/dl included as diabetic. There were 8-10 animals per group in all these studies.

### Vector inoculation

Diabetic animals were inoculated subcutaneously in the footpad of the both hind paws with 30 μl containing 1 × 10^7 ^plaque forming units with either QHmiNa_V _or the control vector QHmiSc 2 weeks after the onset diabetes. Control animals or diabetic only animals were injected PBS in the footpad. A separate cohort of diabetic animals were inoculated with the vector and euthanized 2 weeks later to evaluate the efficacy of knockdown of RNA.

### Cell Culture

DRG neurons from 17-day-old rat embryos were cultured in Neurobasal Medium containing B27, Glutamax I, Albumax I, and penicillin/streptomycin (Gibco-BRL). After 7 days in culture, the cells were transfected with either QHmiNa_V _or QHmiSc at a multiplicity of infection (MOI) of 1 for 2 h. Fresh medium was replaced and collected 7 days later for determination of Na_V_s.

### RT-PCR

cDNA prepared from RNA isolated from DRG cells or rat L4-6 DRG were amplified using following primer sets: β-actin-F (5'-CAG TTC GCC ATG GAT GAC GAT ATC-3') and β-actin-R (5'-CAC GCT CGG TCA GGA TCT TCA TG-3') for β-actin, Na_V_1.6-F (5'-GAC AAT GAT GGT GGA GAC AGA CAC-3') and Na_V_1.6-R (5'-TTG GAG GCC ATC TTT CTG CAG-3') for Na_V_1.6. Na_V_1.7-F (5'-CCA TCA TGA ACG TGC TTC TCG TG-3') and Na_V_1.7-R (5'-CAA AGC AAA GAG CAG AGT GCG GAT C-3') for Na_V_1.7 and Na_V_1.8-F (5'-AAC AGC ACC GGC CAC TTC TTC-3') and Na_V_1.8-R (5'-CCG TTG CTG TTG GGC AGG TTG-3') for Na_V_1.8. All reactions involved initial denaturation at 94°C for 5 min followed by 28 cycles (for β-actin and Na_V_1.7) and 30 cycles (for Na_V_1.6 and Na_V_1.7) at 94°C for 30 sec, 68°C for 3 min, followed by 1 cycle at 68°C for 8 min using a GeneAmp PCR 2700 (Applied Biosystems, Foster City, CA).

### Western Blot

Cells or pooled samples of L4-L6 DRG, were homogenized with lysis buffer (20 mM Tris, pH 7.5, 150 mM NaCl, 1 mM EDTA, 2% SDS, 10% glycerol, and 1:100 dilution of protease inhibitor mixture and phosphatase inhibitor mixture (Sigma), the homogenized cells and tissues were centrifuged at 10,000 × *g *for 10 min at 4°C, and the supernatant was stored at -80°C. An aliquot of supernatant was taken for protein estimation using a protein assay kit (Bio-Rad Laboratories, Hercules, CA). Total cell extract or total protein from DRG (20 μg of protein per lane) was separated by PAGE, transferred to an Immobilon-P membrane (0.45 μm; Millipore), blocked with 5% nonfat milk, and then incubated with the primary antibody. Primary antibodies included an antibody against Na_V_1.7, Na_V_1.8 (Chemicon) and anti-SP-19 (Sigma Aldrich) followed by horseradish peroxidase-conjugated anti-rabbit IgG or anti-mouse IgG (1:5000; GE Healthcare) and visualized with ECL (Pierce) using a PC-based image analysis system (ChemiDoc XRS System; Bio-Rad Laboratories). The membranes were stripped and re-probed with mouse anti-β-actin (1: 2000; Sigma Aldrich) as a loading control. The intensity of each band was determined by quantitative chemiluminescence using a PC-based image analysis system (ChemiDoc XRS System, Bio-Rad Laboratories).

### Immunocytochemistry

Rats were perfused with 4% paraformaldehyde, the L4-6 segment of DRG postfixed and cryoprotected, and 20 μm cryostat sections incubated with anti-GFP (Abcam). The secondary antibodies utilized were fluorescent anti-rabbit IgG Alexa Fluor 488 (1:2000; Molecular Probes, Eugene, OR). Images were captured using a Zeiss LSM 510 Meta confocal microscope.

### In situ hybridization

Rats were anesthetized with ketamine/xylazine (100/10 mg/kg, IP) and perfused with 4% paraformaldehyde in 0.1 M phosphate buffer. L4-6 DRG were post-fixed and cryoprotected overnight at 4°C in 30% sucrose, and serial sections (10 μm) of DRG was cut onto slides and desiccated overnight. Sections from the different groups were processed for *in situ hybridization *for detection of Na_V_1.7 mRNA or GFP mRNA with incubation in 4% paraformaldehyde for 12 min and permeabilization with proteinase K for 6 min followed by hybridization with digoxigenin-labeled probes for Na_V_1.7 (5DigN/TTA CGT CGC CGT CCA GCT CG/3DigN) and GFP (5DigN/TTC TCA TCG TCA CCC TTT TCC T/3DigN) at 53^0 ^C overnight, followed by 1 hr blocking, 2 hrs of Anti-dig-AP antibody incubation and 1 hr color reaction with NBT/BCIP phosphate. The slides were then dehydrated and mounted in Permount. Digitized images of immunostained sections were captured with a Nikon E1000 microscope, and analyzed using a PC-based image analysis program (MCID, Imaging Research, Brock, ON, USA) by a technician blinded to the treatment group. All the cells in the cross-section of the DRG from three animals in each group were analyzed.

## Behavioral studies

### Thermal hyperalgesia

The latency to hind paw withdrawal from a thermal stimulus was determined by exposing the plantar surface of the hind paw to radiant heat using a modified Hargreaves thermal testing device [41]. Rats were placed in individual enclosures on a glass plate maintained at 30°C, and after a 30 min habituation period the plantar surface of the paw exposed to a beam of radiant heat applied through the glass floor. Activation of the bulb simultaneously activated a timer, and both were immediately turned off by paw withdrawal or at the 20 sec cut-off time. Testing was performed by a blinded observer in triplicate at 5 min intervals.

### Mechanical hyperalgesia

Mechanical nociceptive threshold was assessed using an analgesimeter (Ugo Basile, Comerio, VA, Italy) as described by Randall and Selitto [42]. A linearly increasing force was applied through a cone-shaped plastic tip with a diameter of 1 mm onto the dorsal surface of the hindpaw between the third and fourth metatarsals until the rat attempted to withdraw its paw or a pressure of 200 gms reached. The pain threshold determined as the mean of three consecutive stable values expressed in grams was determined by a blinded observer.

### Cold allodynia

Animals were placed on a mesh floor 18 inches above the table and after 20 min of acclimatization, 0.1 ml of acetone was sprayed onto the plantar surface of the hind paw using a 1 cc syringe. The latency of the response, measured as the delay to a withdrawal response of either flinching or licking was used as a measure of cold allodynia, with a cut off limit at 40 sec. A total of 3 responses from each animal were assessed at 5 min intervals by a blinded observer.

### Formalin test

To test inflammatory pain-related behavior, 21 days after inoculation of the vector into one hind paw, normal male Sprague Dawley rats 225-250 grams were acclimated to the test setup and injected with 50 μl of 5% formalin into the plantar surface of the ipsilateral hind paw. The number of flinches over the subsequent 60 minutes counted using an automated device [43].

### Statistical analysis

The statistical significance of the difference between groups was determined by ANOVA (Systat 9) using Bonferroni's correction for the multiple post hoc analyses. All results are expressed as mean ± SEM. All the tissue culture experiments were repeated 3 times. The animal experiments, with 8-10 animals per group, were repeated twice.

## Abbreviations

DRG: Dorsal root ganglia; GABA: gamma amino butyric acid; GFP: Green fluorescence protein; HSV: herpes simplex virus; miRNA: microRNA; Na_V_: voltage gated sodium channel; PDN: painful diabetic neuropathy; STZ: streptozotocin; TTX: tetrodotoxin.

## Competing interests

The authors declare that they have no competing interests.

## Authors' contributions

MC researched data, wrote manuscript. ZZ researched data, reviewed/edited manuscript. SH researched data, reviewed/edited manuscript. MM reviewed/edited manuscript. DJF wrote manuscript. All authors read and approved the final manuscript.

## Supplementary Material

Additional file 1 **Supplementary Figure 1**. In situ hybridization using probe against GFP indicates that a large proportion of the neurons in the DRG were infected by both the miRNA and scramble sequence vectors. Click here for file
